# *Bacillus subtilis*: from soil bacterium to super-secreting cell factory

**DOI:** 10.1186/1475-2859-12-3

**Published:** 2013-01-14

**Authors:** Jan Maarten van Dijl, Michael Hecker

**Affiliations:** 1 Department of Medical Microbiology, University of Groningen, University Medical Center Groningen, Hanzeplein 1, P.O. box 30001, Groningen, 9700 RB, the Netherlands; 2 Institut für Mikrobiologie, Ernst-Moritz-Arndt Universität Greifswald, Friedrich-Ludwig-Jahn-Str. 15, Greifswald, D-17489, Germany

**Keywords:** Bacillus subtilis, Cell factory, Protein secretion, Proteomics, Systems biology, Synthetic biology

## Abstract

The biotechnology industry has become a key element in modern societies. Within this industry, the production of recombinant enzymes and biopharmaceutical proteins is of major importance. The global markets for such recombinant proteins are growing rapidly and, accordingly, there is a continuous need for new production platforms that can deliver protein products in greater yields, with higher quality and at lower costs. This calls for the development of next-generation super-secreting cell factories. One of the microbial cell factories that can meet these challenges is the Gram-positive bacterium *Bacillus subtilis*, an inhabitant of the upper layers of the soil that has the capacity to secrete proteins in the gram per litre range. The engineering of *B. subtilis* into a next-generation super-secreting cell factory requires combined Systems and Synthetic Biology approaches. In this way, the bacterial protein secretion machinery can be optimized from the single molecule to the network level while, at the same time, taking into account the balanced use of cellular resources. Although highly ambitious, this is an achievable objective due to recent advances in functional genomics and Systems- and Synthetic Biological analyses of *B. subtilis* cells.

## 

Industrial enzymes such as proteases, amylases, lipases, and many others are usually produced in Gram-positive bacteria and fungi. These organisms secrete enzymes directly into the fermentation broth, which offers major advantages in their downstream processing. Accordingly, protein export systems play vital roles in enzyme production. With an annual turnover of over 2 billion Euro, this industrial sector has become of substantial economic importance. This is clearly underscored by the production of proteases for detergents, which in Europe alone accounts for the equivalent of up to 900 tonnes of pure enzyme per year. Efficient enzymes are the key to providing alternatives to traditional chemical processes with sustainable biotechnology-based products, for example in the conversion of biomass to biofuel. Importantly, not only in industry, but also in our daily lives, enzymes contribute to sustainability by allowing us to wash clothes at low temperatures. This continuously increasing need for efficient enzymes and their production in a cost-efficient way demands the development of optimized synthetic production organisms.

Gram-positive *Bacillus* species are among the bacterial champions in secreted enzyme production. In biotechnological processes for protein production, *Bacillus subtilis*, *Bacillus amyloliquefaciens* and *Bacillus licheniformis* have become most popular due to their excellent fermentation properties, high product yields (20 to 25 gram per litre) and the complete lack of toxic by-products. *B. subtilis* in particular has been intensely studied over many years and, as a consequence, it is presently the best-characterized Gram-positive bacterium [1]. The strong interest in *B. subtilis* was initially triggered by its simple developmental programs, such as the development of a competent state for DNA-binding and uptake, and sporulation. The high amenability for genetic engineering and large-scale fermentation then made *B. subtilis* an organism of choice for industrial applications [2]. Importantly, the early sequencing of the *B. subtilis* genome represented an enormous technology push [3,4], which was followed up by genome-wide gene function analysis studies [5]. More recently, *B. subtilis* was shown to be an excellent model organism for Systems Biological analyses on gene regulation across many different conditions [6,7]. Such studies were accompanied by extensive proteome analyses that generated a 'panorama view' of the *B. subtilis* proteome and its adaptability to environmental changes and insults [7-9]. These extensive studies over many years have provided deep insights in the mechanisms that *B. subtilis* employs for survival and competitive success in its natural habitats, the upper layers of the soil and plant rhizosphere [1], but they also shed light on the behaviour of this organism during industrial fermentation [6]. Clearly, the high adaptability of *B. subtilis* to environmental changes and insults stands at the basis of its success, not only in nature, but also in commercial applications.

A thorough understanding of proteome dynamics is of crucial importance for biotechnological applications, because proteins are the 'molecular workhorses' of life. Clearly, it is the proteome that translates the blueprint of life - the genome - into cellular processes that can then be harnessed for bioproduction. Importantly, the Systems Biological integration of proteomics with other 'omics' technologies, especially transcriptomics and metabolomics, as well as bioinformatics and mathematical modeling is now leading us to an unprecedented level of understanding of the processes that determine life. Because of their low complexity - only a few hundreds of proteins make up a viable cell - bacteria are the most attractive model systems to study the translation of the genome sequence via the proteins to cell physiology and industrial applications. Here it is noteworthy that, by application of state-of-the-art proteomics technologies, the majority of expressed proteins in *B. subtilis* have already been identified and quantified [8-10]. The picture that emerges from these analyses is that complex mechanisms for the regulation of gene expression guarantee that each single protein is provided in sufficient amounts, at the right time and at the right place (i.e. inside or outside the cell) to organize the essential processes of life. To fully understand these processes and the underlying constraints, it is important that absolute quantitative data - hard numbers of protein molecules per cell - are now becoming available, as is underscored by the fact that most of the cytosolic proteins of *B. subtilis* have been quantified [10; unpublished data]. However, this is just the beginning, because the main challenge that follows is to understand how the hundreds of different protein molecules released from the ribosome organize the life of a bacterial cell. Right after their emergence from the ribosome tunnel, newly synthesized proteins come together to form a huge network of interacting proteins with different levels of complexity. To study and uncover the structure, dynamics and function of this interactome, either as a whole or in parts as represented by protein machines and individual proteins, is one of the major challenges for future research, not only in the fundamental but also in the applied life sciences. Quite clearly, *B. subtilis* appears to be one of the best model systems to tackle this challenge, and this important cell factory's secretion machinery and the network into which this machinery is embedded are logical starting points for in-depth studies.

The fact that many applications of *B. subtilis* are related to the high-level secretion of proteins has focused major research interests on the secretome, which includes both the protein secretion machinery and the secreted proteins [8,11-13]. The analysis of protein secretion in *Bacillus* was initiated in the 1980's with classical molecular genetics approaches, providing valuable insights into cellular components involved in this process [11]. However, the classical approaches did not provide global insights into the importance of individual components for the overall flow of proteins from the cytoplasm to the membrane, cell wall and extracellular environment. This required the application of proteomics techniques, which was pioneered with protein secretion studies in *B. subtilis* that, in fact, introduced the term 'secretome' for the very first time in the year 2000 [12-14]. The combined proteomics and molecular biological analyses showed that *B. subtilis* secretes proteins for a wide range of purposes, including nutrient acquisition, competition with other organisms and adaptation to changes in the environment [8,12,13,15]. Importantly, proteomics helped defining the composition of the extracellular proteome (exoproteome), the cell wall proteome and the membrane proteome, showing that these sub-proteomes of the cell are highly dynamic entities that change in response to growth stage, nutrient availability and stresses, not in the last place the 'secretion stress' caused by the overproduction of secretory proteins [13,16,17]. While the initial secretome analyses were carried out by two-dimensional gel electrophoresis and subsequent Mass Spectrometric analyses [12], more recent studies involved gel-free techniques that actually allow the detection and often also the quantification of all known secretion machinery components and secreted proteins [8,10; unpublished observations]. A very important outcome of the exoproteome analyses was the definition of secretion signals, the so-called signal peptides, for all secreted proteins of *B. subtilis*[8,12,13,18]. This has paved the way for current Synthetic Biology approaches in which the most effective signal peptides for the secretion of particular proteins of interest are selected from the global library of *Bacillus* signal peptides [19].

The secreted proteins of *B. subtilis* usually contain N-terminal signal peptides that are important for export from the cytoplasm. These signal peptides are composed of three characteristic domains generally known as the N-, H- and C-regions [13,20]. Arginine or lysine residues in the N-region interact with the translocation machinery and negatively charged phospholipids in the cell membrane. The H-region comprises hydrophobic residues that adopt an alpha-helical conformation, which is necessary for the initiation of protein translocation across the membrane. The C-region contains the recognition and cleavage sites for signal peptidases (SPases) that cleave off the signal peptide to release the mature protein from the membrane during or shortly after translocation [21]. Cleaved signal peptides are then targeted for degradation by the ‘site-2’ membrane protease RasP, and possibly other membrane-associated proteases [20-22].

Importantly, the signal peptides of secreted proteins are specifically recognized by dedicated protein translocation machineries. The most relevant and best-studied translocation pathways for secretory proteins in *B. subtilis* are the general secretory (Sec) pathway and the twin-arginine (Tat) translocation pathway [11,16]. Both pathways are highly conserved throughout all kingdoms of life. Signal peptides that target proteins to the Tat pathway contain a so-called twin-arginine (RR-)motif, and they are generally less hydrophobic than signal peptides that target proteins into the Sec pathway [13,23]. The majority of secreted proteins are translocated in an unfolded state from the cytoplasm to the exterior of *B. subtilis* via the highly conserved Sec pathway. This involves (i) cytoplasmic chaperones that maintain a translocation-competent unfolded state of the secretory precursor proteins, (ii) the Sec translocase consisting of the SecA translocation motor and the membrane-embedded channel proteins SecY, SecE, SecG and SecDF, (iii) SPases for removal of signal peptides from translocated proteins, and (iv) catalysts of post-translocational protein folding, like the chaperone and peptidyl-prolyl cis/trans isomerase PrsA and the thiol-disulfide oxidoreductases BdbB, BdbC and BdbD. Lastly, the translocated and fully folded proteins need to traverse the cell wall in order to be secreted into the external milieu. The cell wall contributes to the post-translocational folding of secretory proteins, because it is a major reservoir of metal ions that are required for the folding, stability and activity of many secreted proteins [24].

The Tat pathway is less well defined. It essentially consists of two components named TatA and TatC. A TatAC complex has been proposed to serve as the receptor for RR-signal peptides [25]. The TatC subunit then drives the membrane insertion of the signal peptide [26,27], and membrane translocation of the full precursor protein requires the association of the TatAC-precursor complex with additional oligomeric TatA complexes [25]. Intriguingly, while TatAC complexes represent the minimal translocases, the situation in *B. subtilis* is somewhat more complex as the TatA subunits have been triplicated, while the TatC subunits have been duplicated [25]. Recent studies indicate that 'mixed translocases' including multiple Tat subunits may be active in *B. subtilis*[28-30]. While the *B. subtilis* Sec pathway is successfully employed for the production of heterologous secreted proteins, the Tat pathway of this organism remains to be unlocked for this purpose [31,32]. Opening the Tat pathway for secretory protein production is an interesting and potentially highly rewarding challenge for cell factory engineering since this pathway can secrete proteins that have been fully folded in the cytoplasm, including enzyme complexes and proteins with bound cofactors [31,32]. The *B. subtilis* Tat machinery is intrinsically capable of this astonishing feat, but this is only evident when it is heterologously expressed in *Escherichia coli*[33,34]. In *B. subtilis* itself, this pathway is highly selective in the acceptance of substrates and the codes for this selectivity remain to be cracked [32,35]. Possibly, the key for opening Tat is to be found within the complex interaction network of which the Tat subunits are part. This network was recently shown to include several novel factors that are required for Tat-dependent protein translocation [30].

How can we transform the already highly efficient *Bacillus* cell factory of today into the next-generation super-secreting cell factory that we need tomorrow? We believe that this will require integrated Systems and Synthetic Biology approaches that address the *Bacillus* secretion machinery from the level of single molecules, to the levels of protein complexes and interaction networks up until the metabolome level and whole-cell physiology. At the single-molecule level we need to understand better how individual translocase components insert into the membrane. Next, we need to find out in detail how these molecules interact with each other, not only within the plane of the membrane, but also with the cytoplasmic targeting factors, and the extracytoplasmic SPases and folding catalysts. Where possible, these interactions need to be optimized so that protein targeting, translocation and folding are no longer limiting steps in secretory protein production. Not in the last place, we need to understand how secretory precursor proteins interact with these secretion machinery complexes. Here, it is of relevance to find out why different signal peptides that, in essence, all fulfil the same targeting roles can make such a huge difference in the efficiency of protein secretion [19,36,37]. Clearly, this must relate to differences at the single molecule levels that impact on the efficiency of interactions at the translocation complex level. Importantly, synthetic biological principles have already been applied to define the best-possible signal peptides for particular heterologous protein secretion [26,27], and investigations of this type are likely to lead to the elucidation of the principles that govern signal peptide function in detail. Furthermore, as underscored by recent studies on the Tat interactome [30,38], the larger interaction networks in which the secretion machinery is placed must be better understood for rational secretion machinery engineering according to Synthetic Biological principles. In this context, we also need to pinpoint the beneficial and detrimental activities of the cellular protein quality control machinery, such that the beneficial activities are enhanced while the detrimental ones are minimized or even completely eliminated [39]. This focuses a strong interest on proteases that are active within the plane of the membrane or at the cytoplasmic or extracytoplasmic membrane surfaces [20,24]. Such proteases have important roles in the removal of potentially detrimental molecules, such as cleaved signal peptides, incompletely synthesized proteins and malfolded proteins. However, some membrane proteases may also be 'overactive', which can lead to important product loss. Therefore, the machinery for quality control needs to be fine-tuned with the actual need for quality control, so that product losses are minimal while product quality remains optimal. Achieving these goals will require substantial investments in terms of fundamental and applied research, but several secretion machinery engineering approaches have evidenced that there is ample room for further improvements of the *Bacillus* cell factory as it is today. For example, the yield of human interferon-α2b was shown to be increased in a strain with an engineered SecA translocation motor [40]. Also, SecA was engineered such that the *E. coli* targeting chaperone SecB could be exploited for demonstrably improved protein secretion in *B. subtilis*[41]. Other studies showed that engineering of SPases [21] and post-translocational protein quality control factors like PrsA and thiol-disulfide oxidoreductases would lead to substantial increases in protein production [24,42-44], albeit sometimes at the expense of product quality [34]. In line with these findings, also the elimination of multiple extracellular proteases that degrade slowly folding proteins or folded proteins that expose protease cleavage sites can lead to substantial improvements in productivity [45-51]. This is not only relevant for proteins that are secreted via the Sec pathway, but also for proteins secreted via the Tat pathway [52].

As exemplified by studies on the improved export of disulfide bond-containing proteins in *B. subtilis*, where lowering the reducing power of the cytoplasm by depletion of the thioredoxin A was found to be beneficial [44], cell factory engineering approaches should not only address the components of the protein secretion machinery, but also the general cell physiology. Here, the latest advances in the Systems Biological analysis of cellular responses to changing conditions, proteomics in particular, will probably be of enormous value. This view is perhaps best supported by recent studies on the adaptation of *B. subtilis* cells to nutritional shifts between two preferred carbon sources, glucose and malate, which requires dynamic interactions between metabolic and regulatory networks [7]. To understand these responses, the dynamic transcript, protein, and metabolite abundances and promoter activities were determined and subsequently analysed through combined statistical and model-based approaches. Interestingly, these analyses revealed that complex adaptive changes in interactions across multiple levels of regulation can also be achieved by controlling single genes. This implies that, in principle, the same Systems Biological methodology can now also be applied to control the cellular responses to high-level protein production and secretion. This would require a quantitative multi-omics analysis of protein production- and secretion stress responses, followed up by rigorous statistical analyses and mathematical modeling. Based on the outcomes, it should then be possible to reduce, or even eliminate, the major stress responses that are triggered by large-scale protein expression and secretion, possibly by controlling only a few genes that minimise the adverse effects of protein production and secretion stress and, at the same time, enhance the positive effects. A most powerful approach would also take into account the management of cellular resources and growth rate [53-58], so that the *Bacillus* cell factory can be optimally geared towards the production of secreted proteins. While this may sound very ambitious, the technology and mathematical tools for such a cell factory engineering exercise are already in place today. This is exemplified by genome engineering approaches, which were initiated with the relatively simple removal of prophages, prophage-like regions, and a large dispensable operon from the *B. subtilis* genome [59]. Subsequently, a large-scale genome minimization effort was undertaken, yielding a strain (MBG874) that lacks 874 kb (20.7%) of the *B. subtilis* genome [60]. Importantly, this minimized strain displayed an improved productivity for secreted enzymes, including a thermostable alkaline cellulase, an alkaline protease, and an alkaline α-amylase [60-62]. Very recently, a systematic mapping of non-essential regions in the *B. subtilis* genome was undertaken based on the model-driven design of dispensable intervals [63]. This rational approach has yielded a collection of 286 deletion mutants lacking large dispensable genomic regions that, altogether, cover ∼ 76% of the genome. The knowledge gained from these studies can now be applied to engineer next-generation *Bacillus* cell factories with customized genomes and stream-lined metabolism.

Lastly, for obtaining maximum yields, the production of proteins of interest needs to be fine-tuned with the available cellular resources. Physiological proteomics is a particularly promising approach to achieve this goal. Here, the establishment of a proteomic stress and starvation signature library, which pinpoints the proteins strongly induced in response to different stress and starvation stimuli, will facilitate the design of strong and auto-regulatable promoter structures that respond adequately to the imposed stress/starvation stimuli [64-67]. Such engineerd promoters can then be implemented as 'biobricks' in smart expression cassettes that also include optimal signal peptides for super-secretion of the desired product.

In conclusion, the efficient production of secreted enzymes by microorganisms like *B. subtilis* is one of the key drivers of today's successes in the enzyme industry. To achieve even higher production yields, it is crucial to pinpoint and remove the current production bottlenecks. Based on recent progress in fundamental and applied *B. subtilis* research, combined Systems and Synthetic Biological approaches can now be harnessed for the construction of next-generation super-secreting *Bacillus* cell factories.
